# Integrative care for the management of low back pain: use of a clinical care pathway

**DOI:** 10.1186/1472-6963-10-298

**Published:** 2010-10-29

**Authors:** Michele J Maiers, Kristine K Westrom, Claire G Legendre, Gert Bronfort

**Affiliations:** 1 Northwestern Health Sciences University, 2501 W 84th St Bloomington, MN 55431, USA

## Abstract

**Background:**

For the treatment of chronic back pain, it has been theorized that integrative care plans can lead to better outcomes than those achieved by monodisciplinary care alone, especially when using a collaborative, interdisciplinary, and non-hierarchical team approach. This paper describes the use of a care pathway designed to guide treatment by an integrative group of providers within a randomized controlled trial.

**Methods:**

A clinical care pathway was used by a multidisciplinary group of providers, which included acupuncturists, chiropractors, cognitive behavioral therapists, exercise therapists, massage therapists and primary care physicians. Treatment recommendations were based on an evidence-informed practice model, and reached by group consensus. Research study participants were empowered to select one of the treatment recommendations proposed by the integrative group. Common principles and benchmarks were established to guide treatment management throughout the study.

**Results:**

Thirteen providers representing 5 healthcare professions collaborated to provide integrative care to study participants. On average, 3 to 4 treatment plans, each consisting of 2 to 3 modalities, were recommended to study participants. Exercise, massage, and acupuncture were both most commonly recommended by the team and selected by study participants. Changes to care commonly incorporated cognitive behavioral therapy into treatment plans.

**Conclusion:**

This clinical care pathway was a useful tool for the consistent application of evidence-based care for low back pain in the context of an integrative setting.

**Trial registration:**

ClinicalTrials.gov NCT00567333

## Background

When addressing low back pain (LBP), there appears to be no one treatment that is best for all patients. Instead, several viable treatment options exist, including many complementary and alternative therapies[1-3]. Despite this, the treatment of LBP remains a major challenge to the healthcare system, both in terms of effective management and cost. Owing to the complexity and multidimensional nature of LBP, it is plausible that a combination of efficacious treatments, based on an individual's presentation, could exceed the therapeutic effect of any one of these therapies alone[1-4].

Several models of integrative care have been described with varying levels of collaboration between providers[5]. While conventional medicine has enhanced efforts to integrate healthcare services within allopathic disciplines,[6] the combination of conventional medicine with complementary and alternative medicine (CAM) has not been extensively explored. It has been proposed that an optimal integrative model of care should involve a collaborative, interdisciplinary, and non-hierarchical team approach. By combining the efforts of multiple providers, it is hypothesized that this collective effort can exceed what can be accomplished by monodisciplinary care, particularly for chronic conditions[4,5,7-9].

Further, it has been demonstrated that when patients participate in their care, they tend to be more satisfied and experience better outcomes[10]. Multiple efficacious treatments introduce greater opportunity for patient choice. Patient context and centrality have been identified as crucial indicators of successful integrative care[11]. Therefore, partnerships between CAM and conventional medical providers should be extended to actively include patients as a means of providing a best practice approach to decision-making and patient-centered care.

To date, there are few studies of integrative care for the management of LBP. One study suggests that providing individualized treatment within a multidisciplinary, conventional medicine setting results in faster return to work for chronic LBP patients[12]. The inclusion of efficacious CAM therapies in such an environment has the potential to enhance outcomes and warrants further research. To be successful, this type of integration requires methods for delivering coordinated, evidence-based decisions that can take place in a variety of clinical settings[6]. To improve quality and outcomes, it is important to emphasize the processes of care which prioritize inter-provider cooperation, increased transparency, and unfettered flow of information.

Clinical pathways have been defined as structured, multidisciplinary plans of care designed to support the implementation of protocols and clinical management[13]. These management tools are often defined for a specific group of patients in which interventions by healthcare professionals are optimized and monitored using outcome measures. Care pathways can serve as vehicles for the efficient application of evidence-based healthcare to provide consistent yet personalized high quality care[14,15]. Pathways may also facilitate integration among multiple provider types, enabling a common language and shared perspective of the patient's entire care process[16]. The process used by a multidisciplinary group of providers to provide care for LBP patients has not been well documented in the scientific literature. This paper describes the use of a care pathway designed to guide clinical decision making among an integrative group of providers within a randomized controlled trial for LBP.

## Methods

### Study Context

The authors and their investigative team are completing a randomized clinical trial (Trial Registration NCT00567333) to compare the clinical and cost-effectiveness of individualized treatment for LBP in either a monodisciplinary chiropractic or multidisciplinary integrative care setting. Individuals with chronic LBP ≥ 6 weeks in duration were randomly assigned to receive 12 weeks of either monodisciplinary chiropractic care or multidisciplinary integrative care. A 12 week intervention period was perceived by study clinicians and investigators to be typical when treating this population. Integrative care consisted of acupuncture and Oriental medicine (AOM), chiropractic (DC), cognitive behavioral therapy (CBT), exercise therapy (ET), massage therapy (MT), medication (Med), and self-care education (SCE), provided either alone or in combination. Providers in each intervention group formed a clinical care team (a monodisciplinary chiropractic team and a multidisciplinary integrative team). Approval was granted by the Institutional Review Boards of collaborating organizations; written informed consent was obtained from all participants. A detailed description of the methods, protocols, and interventions used in this study has been reported[17].

### Care Pathway

**Figure 1:**
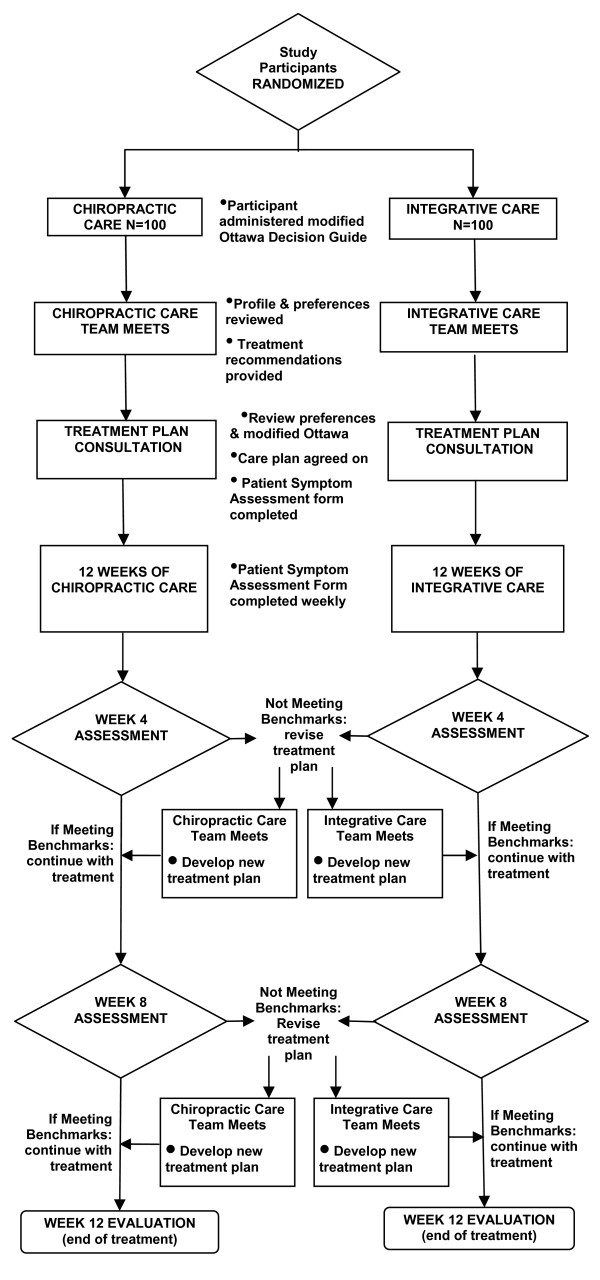
**Care Pathway**.

Both clinical care teams were guided by a clinical care pathway, designed to standardize the process of developing recommendations and delivering treatment to study participants (see Figure 1). This paper will focus on the care pathway used by the multidisciplinary integrative team.

Recommendations for treatment were based on an evidence-informed practice model, which included the participant's clinical presentation at baseline evaluation, their expressed values and expectations, the best available scientific evidence, and the team's clinical experience. Clinicians were guided by general principles and goals. These included individualizing treatment as per each study participant's unique presentation, while creating plans that minimize fear and catastrophizing, emphasize active care, decrease dependency on the healthcare system, consider patient preferences as well as cost-effectiveness, and avoid arbitrary limits to care.

### Selected Modalities

Both conventional and complementary modalities were included as part of integrative management. A search of the literature was conducted to identify systematic reviews and evidence-based guidelines of non-surgical interventions for back pain. Therapies were considered for this model of care if there was evidence to support their use for chronic LBP, or in the absence of evidence indicating harm or ineffectiveness[18-28]. Additionally, these therapies must have been considered typically viable and accessible options to the study population. Named, proprietary techniques were excluded because it was felt they were not uniformly available in the U.S. Herbs and nutraceuticals were also not permitted in this study due to the investigators' inability to uniformly guarantee the quality and potency of these products. Credentials and appropriate licensing of all treatment providers were verified prior to the study in addition to extensive training in human subjects' protection and study protocols.

Seven therapies, provided by six types of providers, were chosen for inclusion in this integrative model, based on scientific evidence and what is considered typical for conservative non-operative care in the U.S. Acupuncture has been shown in a Cochrane review to be more effective for pain relief than no treatment or sham, and more effective for improving function in the short-term[24]. Cognitive behavioral therapy was found in to reduce pain and improve behavioral outcomes when compared to placebo or no treatment[21]. Exercise, particularly individually designed and supervised programs that focus on high dose stretching and strengthening maneuvers, has been shown to be effective for LBP sufferers[22]. Massage therapy reviews, combining evidence from higher quality recent trials, has demonstrated evidence of overall benefit and some pain relief lasting up to a year[23]. Medication, a common conventional approach to LBP management, has evidence of effectiveness supporting short-term use of NSAIDS and weak opioids for pain relief, as do antidepressants, muscle relaxants, and capsicum plasters[18,19]. Self-care education, or information designed to improve health behaviors relative to the management of LBP, is supported by the evidence, particularly when focused on improving patients' understanding of LBP, reducing unwarranted concern, and fostering a sense of empowerment[20]. Spinal manipulation therapy, delivered in this study by chiropractors, has been found to be effective when compared to sham, and produce outcomes comparable to other efficacious therapies[26,27].

### Team Training

Training of the care team was led by a consultant with expertise in the development of healthcare teams and group dynamics. A series of workshops and written materials were used to train clinicians to apply study protocols, the principles of evidence-based healthcare, and how to achieve non-hierarchical group consensus. These were revisited as part of an ongoing quality assurance plan and if deviations from desired protocol were observed. Of note, it became apparent within the first few months of the study that further training in the interpretation of the biomechanical and orthopedic tests would benefit team members with less background in Western diagnosis. In addition to reviewing the scientific evidence substantiating the use of these biomechanical and orthopedic tests, the team toured the biomechanical testing lab to observe how measures of strength, endurance, and range of motion were obtained. Additionally, some members were less familiar with the patient self-assessment measures; these were also reviewed.

The consultant trained case managers to lead care team meetings, facilitate discussion, and maximize group dynamics. This included how to keep meetings consistent using a regular format, as well as how to engage all members of the care team to contribute to group decisions. These case managers were also responsible for relaying treatment plan recommendations, agreed to by the clinical care team, to respective study participants.

Initial training began with an all-day workshop the month prior to enrollment of study participants. During the workshop, information about the prevalence of chronic LBP was presented to provide the clinicians with context for the trial. Next, each modality was addressed by a representative member of the team who explained the background and history of their discipline. Research investigators then gave an overview of the available evidence which supported the use of each modality in the treatment of chronic LBP. The concept of evidence-informed practice (EIP) was defined for the team as the combination of the best available evidence, the clinician's experience, and the patient's preferences. The team was asked to use the principles of EIP when making clinical decisions in the trial.

Clinicians were shown how to create treatment plan recommendations based on a common patient profile. After enrollment, an examining clinician created a patient profile (see below) to describe the clinical characteristics of a participant, which summarized the history, exam, psychometric tests, biological measures, and treatment preferences recorded at the baseline evaluation. If randomized to the integrative care arm of the trial, that participant's patient profile was forwarded onto the integrative care team and formed the foundation for creating treatment plan recommendations. Training focused on helping the clinicians interpret the profile in order to devise individualized recommendations. The team was also trained in the mechanics of the weekly team meetings, including implementing guiding principles when designing treatment plans, achieving consensus by multi-voting, and adhering to best practice concepts. The study consultant conducted a series of site visits to observe team dynamics, and provide feedback and additional training as necessary.

### Patient Profile

A profile was created on each randomized participant and was meant to provide clinicians with a uniform, comprehensive assessment of the presenting complaint from a bio-psycho-social perspective. Information used in the profile was obtained during the baseline evaluation prior to randomization, during which time self-assessment questionnaires and a clinical evaluation were conducted. The profile consisted of participant information with regards to LBP and health history, examination, imaging, psychosocial measures, preference and previous experience, objective biomechanical assessment, and a classification of the LBP according to the Quebec Task Force (see Table 1)[29].

**Table 1:** Information in patient profile

History	• Activity and employment status
	• Description of LBP
	• Response to previous treatment
	• Co-morbidities and medications
Examination	• Vitals (height, weight, blood pressure)
	• Orthopedic exam (Lasegue, Patrick Fabre, Kemp, Gaenslen, Femoral Nerve Stretch)
	• Neurologic exam (motor, sensory, reflex evaluation)
	• Soft tissue evaluation

Imaging	• X-ray results (obtained if clinically warranted)
	• DEXA results (obtained if clinically warranted)

Biomechanical Assessment	• Torso strength (flexion, extension)
	• Torso endurance (flexion, extension, lateral bridge)
	• Lumbar range of motion (flexion, extension, lateral bending, rotation)

Psychosocial Assessment	• Disability (modified Roland Morris)[32,33]
	• General health status (EuroQol) [34,35]
	• Fear avoidance (Fear Avoidance Beliefs Questionnaire)[36]
	• Kinesiophobia (Tampa Scale of Kinesiophobia)[37]
	• Coping style (Vanderbilt Pain Management Inventory)[38,39]
	• Pain self-efficacy (PSEQ)[40]
	• Depression (CES-D)[41,42]

Preferences	• Which modalities were preferred
	• Expectations for improvement with each modality (5 point scale)

### Reaching Consensus

Weekly meetings, facilitated by case managers, were held. Clinicians in the integrative care team prepared beforehand by reviewing the profiles of newly randomized participants and devising a treatment plan to recommend to the group. During the team meetings, each clinician was asked to present their suggested plan, as well as a rationale for their selection. Once each member contributed, group discussion was facilitated by a case manager to explore the recommendations. Finally, the most well-liked treatment plans were nominated by team members to be put to a vote.

Voting used the "fist to five" technique: five fingers represented "I strongly agree," three fingers "I can live with that," and one finger "I strongly disagree." A fist meant the topic required more discussion, after which the vote was again called. Any vote that did not elicit three fingers or greater by each team member failed to reach consensus and was not brought forward as a recommendation. This process was repeated until at least one treatment plan gathered consensus and no additional treatment plans were identified and called on to vote. Case managers documented group discussion and consensus decisions during this process.

### Treatment Plan Consultations

Recommended treatments were subsequently presented to the study participant during a treatment plan consultation with the case manager. The various treatment plan combinations and their accompanying rationales were explained and participant questions were answered. When the participant demonstrated a fully informed comprehension of the treatments being offered to them, they selected a care plan, and 12 weeks of treatment with the selected modalities were scheduled.

### Treatment Management

As a means for clinicians and participants to monitor clinical change during study treatment, the Patient Symptom Assessment Form (PSAF) was designed, using the Measure Yourself Medical Outcome Profile (MYMOP)[30] as a template. Modifications which made the PSAF more useful in this trial included instructions specific to an individual with LBP and an 11-point ordinal scale similar to those the participants completed throughout the study on patient self-report questionnaires. The PSAF was not an outcome measure, but rather provided treating clinicians with a tool to objectively assess changes in clinical outcomes that were most important to the study participant. The trend in these changes over the course of care served as a formalized guide to the clinician to modify the treatment plan as necessary.

During the treatment plan consultation, participants were asked to choose the symptom, physical or mental, most bothersome in regard to their low back condition and rate its severity over the past week on a scale of 0-10 (0 = "as good as it could be," 10 = "as bad as it could be"). Similarly, they chose an activity that their low back problem makes difficult or prevents them from doing and rated it in a similar fashion. These two outcomes were rated by the participant on a weekly basis prior to their treatment visit. Clinicians reviewed the responses according to the clinical care pathway after four and eight weeks of treatment, and compared them to benchmarks for expected improvement.

### Benchmarks

Benchmarks for improvement were generated from clinical data previously collected by this research team. Global improvement ratings at weeks 4 and 12 were used to set standards for expected improvement. Although it is unknown how global improvement scores compare to a participant's self-selected symptom and activity rating, they were assumed to be similar for the purpose of setting benchmarks. Clinicians were expected to bring the participant's case back to the care team for review and consider altering the treatment plan if benchmarks for improvement were not met. Other triggers which brought a case back for discussion included a worsening of the participant's LBP or if the clinician or participant were dissatisfied with the care process. In these cases, the profile was again presented and treating clinicians shared their observations. Consensus voting occurred if there was a recommendation to alter the treatment plan; corresponding modifications to the treatment plan were discussed with the study participant and implemented.

Communication between providers, especially when the participant was co-managed between providers, was facilitated by case managers, project managers, shared access to treatment notes, and encouraged during weekly meetings. Discussions or decisions regarding care were documented and maintained in treatment files.

## Results

The integrative care team was comprised of 13 licensed or certified providers, including 3 acupuncture and Oriental medicine (AOM) providers, 2 chiropractors (DC), 2 cognitive behavioral therapists (CBT), 2 exercise therapists (ET and SCE), 3 massage therapists (MT), and 1 medical physician (MED). Care provided by disciplines that are not licensed in the U.S. was supervised by the licensed medical physician and chiropractors. Each care team meeting was led by two of the three case managers, trained as facilitators. Treatment plans recommended by the team were presented to study participants by one of the three case managers.

Observations by the expert consultant on healthcare team dynamics observed that the integrative care team transitioned early into a high performing group. Members arrived to meetings prepared, were respectful in communication, and operated with an eye toward accomplishing designated goals. The facilitators assisted the team by setting consistent agendas, managing time during the meetings, and providing feedback to the group when it was warranted (e.g. reminders of guiding principles). The consultant also observed that the team appeared to value diverse perspectives, exemplified when members suggested treatment options from other disciplines, suggesting learning and cross-knowledge within the group.

**Figure 2:**
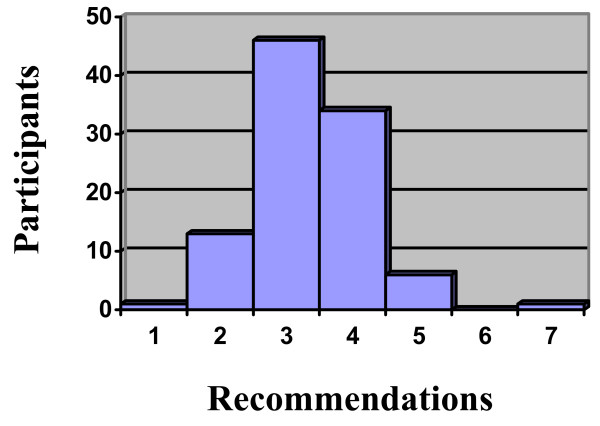
**Number of recommendations offered to participants**.

A total of 201 patients were randomized to the study; of these, 101 were randomized to receive integrative care. The number of treatment plans offered to a participants in this arm of the study ranged from 1 to 7, with a mean of 3.4 and mode of 3.0 (see Figure 2). There were typically two or three different treatment modalities per treatment plan option, with the most common being MT+ET+SCE, AOM+ET, and AOM+ET+SCE (see Table 2). These were also the most frequently selected treatments by study participants (see Table 2). ET, SCE, AOM, and MT were commonly offered modalities by the group, and also regularly selected (see Table 3). In addition to preference, high expectations for improvement, and previous success with treatment, other recurring rationales given by providers for offering each modality are listed in Table 4.

**Table 2:** Most common treatment plan recommendations

Treatment Options	Recommended	Selected
MT+ET+SCE	39	16

AOM+ET	37	13

AOM+ET+SCE	36	23

MT+ET	30	6

DC+ET	26	7

DC+ET+SCE	21	5

MT+CBT+ET	14	9

AOM: acupuncture and Oriental medicine; CBT: cognitive behavioral therapy; DC: chiropractic; ET: exercise therapy; MT: massage therapy; SCE: self-care education

**Table 3:** Use of individual modalities, alone or in combination with other modalities

Modality	^**1**^**Offered Modality**	^**2**^**Selected Modality**	^**3**^**Frequency Selected**	^**4**^**Received Modality**
ET	95	90	94.7%	96

SCE	74	57	77.0%	59

AOM	71	49	69.0%	51

MT	71	39	54.9%	37

DC	56	17	30.4%	19

CBT	37	18	48.6%	35

Med	3	3	100.0%	5

AOM: acupuncture and Oriental medicine; CBT: cognitive behavioral therapy; DC: chiropractic; ET: exercise therapy; MT: massage therapy; Med: medication; SCE: self-care education

1. Number of participants offered this modality during their initial treatment plan consultation

2. Number of participants who selected this modality during their initial treatment plan consultation

3. Percent of time participants selected the modality offered to them during their initial treatment plan consultation

4. Number of participants who received the modality at any point during the 12-week intervention phase

**Table 4:** Common rationales for recommendations

Modality	Common Rationales
Exercise	• Objective biomechanical testing suggest need for
	- strengthening, endurance training
	- increased flexibility
	• Fear of movement or activity

Self Care Education	• Aggravating factors could be addressed with
	- ergonomics
	- activity modification

Acupuncture & Oriental Medicine	• Stimulate flow of energy (qi) to decrease pain
	• Presentation fits classic Oriental medicine pattern

Massage	• Tight muscles noted on exam/history
	• Benefit from a touch therapy; stress reliever

Chiropractic	• Reduce pain
	• Increase joint range of motion

Cognitive Behavioral Therapy	• Need skills for relaxation or activity scheduling to manage pain
	• Address fear of movement

Medication	• Pain management
	• Review current medication

Only one study participant randomized to integrative care did not agree to any of their treatment recommendations; in this instance, the case manager reached a compromise between the participant's preference and the care team's recommendation and returned the case to the care team for a revised treatment plan.

Of 101 individuals, the treatment plans for 38 were re-evaluated over the course of care by the integrative team; two participants were discussed on multiple occasions (see Table 5). The most frequent reasons that cases were brought back to the care team included a lack of improvement as perceived by the provider, patient, or both. Established benchmarks for improvement proved to be a useful, objective tool for identifying cases where a change in treatment could be warranted. Additionally, they provided patients with a more tangible rationale for the clinician's desire to change treatment plans. Occasionally, additional information was divulged by the patient during treatment, resulting to a change in the treatment plan. The most common changes made included the addition of CBT (17/36), AOM (7/36), and ET (6/36).

**Table 5:** Re-assessed cases

Decisions from Integrative Team	N = 38
Resulted in change to treatment*	34

Added a modality	28

Replaced a modality	8

Discontinued a modality	1

No change	4

*2 participants had changes made to their treatment plan on more than one occasion

## Discussion

The care pathway designed for this trial proved to be an essential mechanism for operationalizing a model of integrative care. Providing the care team with a deliberately outlined process to follow, supported by standardized patient information and guiding principles, allowed them to consistently and effectively apply treatment plans. Additionally, the care pathway functioned as a detailed quality assurance system, which was useful in maintaining the integrity of study methodology across varied treatments. It is unlikely that the integrative team of providers could have provided consistent care without the structure provided by the care pathway; case managers were integral to reinforcing this process. The pragmatic design of this research study required a high level of communication and flexibility between participants, providers, case managers, and project managers. This is particularly true considering that one-third of participants receiving treatment from the integrative team were re-assessed over their course of care.

There are several potential explanations for what could be considered a large proportion of patients whose care plans were revised by the integrative team. It is possible that what were perceived by the care team as "optimal" treatment plans were not effective. Also, the benchmarks that served as a trigger for considering other treatment options may not have allowed adequate time for the intervention to result in improvement. This is complicated by the chronic and episodic nature typical of LBP, for which a 12 week treatment period may not be ideal. Identifying an optimal intervention period and benchmarks, and furthermore tailoring them to the individual, will require extensive future research. Alternatively, it could be argued that treatment for any chronic condition should remain fluid, and that making changes to a care plan is a key characteristic of maximizing clinical outcomes. As the study is ongoing, clinical outcomes in this sample have yet to be analyzed.

Steps taken in the design and evaluation of the integrative care group in this trial is consistent with the recommended framework set forth for the study of complex interventions[43]. A "pre-clinical" phase explored the literature on treatments and integrative care models for LBP, for use as the theoretical basis to construct an optimal integrative care team. Modeling, or Phase I, explored best practices in clinical care to delineate components of care and inter-relationships amongst providers that could affect outcome. These were used to set parameters around clinical decision-making for the group and became shared guiding principles for treatment. The trial itself was a hybrid of Phases II and III, with attention paid to features of well designed clinical trials. The integrative arm, however, remained somewhat fluid in terms of type, frequency, and delivery of care, creating an intervention that could adapt to the dynamic and iterative needs of participants.

Taking patients' preferences into account during study treatment was intended to reflect best practices in care and approximate the role choice plays in clinical practice. The impact this had on clinical outcomes will be assessed in future study analysis through expectation and satisfaction questionnaires. Additionally, qualitative interviews were conducted to capture various aspects of patients' experiences with the clinical encounter, including what they liked and did not like about their care. These results will be considered when assessing the "success" of this integrative model, as well as informing modifications to the care pathway for use in clinical practice and future research study design.

The integrative care team continued to evolve over the course of the study, moving through recognized stages of team building: forming, storming, norming, and performing[31]. While changes in group dynamics may be considered a limitation to the study design, it is inherent to any team; therefore, it is important to regularly monitor and address these changes throughout the life of the group. The integrative care team transitioned early into a "norming" phase of team building, and reached a point where they were comfortable recommending each other's therapies. Team members learned early how to strike a balance between being the "expert" with regard to their own disciplines and remaining open to, at times, conflicting opinions of other healthcare professionals. This contrast occasionally created a healthy tension but was managed with open exploration and discussion among team members under the guidance of the group facilitator. Conversely, group members' motivation to reach consensus may have resulted in a reluctance to disagree. Some team members appeared to be highly focused on harmony, at the expense of engaging debate and risking group discord.

Relatively few study participants selected medication as a preferred or desired treatment option. There are several possible explanations why individuals tended toward selecting acupuncture, massage, and exercise interventions instead. This study was conducted at a university that focuses on complementary and integrative healthcare; this context may have been seen as a safe and credible environment to try treatments outside of mainstream healthcare. Further, the study was an opportunity to receive treatments at no cost. Additionally, many participants were currently taking or had already taken medication for their LBP and may have felt that a medication consultation would not provide any additional help. The side-effect profiles of commonly used pain medication could have also discouraged participants from making this selection. Chiropractic care was also not commonly recommended to, or selected by, study participants randomized to the integrative care arm of the study. Considering chiropractic care alone was the comparison group treatment, it may have been seen as not "different enough" to use as a treatment option in the integrative care arm. Similar to medication, participants may have been interested in trying other complementary treatments that are considered to be more "alternative" to mainstream healthcare.

Cognitive behavioral therapy was the modality most often declined at the initial treatment consultation. Interestingly, it also was the most commonly added modality if additional care was determined necessary during the intervention phase of the study. The integrative care team often felt that participants who had been previously wary of CBT eventually became amenable to it, once a primary relationship with another clinician had been established.

The model of integrative care created in this study was based on identification of effective treatment options through a review of the research literature, and consideration of what therapies are typically accessible treatment options in the context of the study population. Most of the chosen treatment options were readily available at the site of the trial, located at a health sciences university clinic with access to complimentary and alternative healthcare practitioners, as well as a medical doctor. Two licensed clinical psychologists were added to the team to provide cognitive behavioral therapy.

A limitation of this study is the question of its generalizability. This idealized care pathway was created by study investigators to optimize a collaborative, non-hierarchical design. It operated outside the context of time, resource, and financial restraints that are practical realities of most healthcare environments. Further, this study was conducted in the U.S., where the delivery system and certification of providers is often different from other countries. Access to various types of care modalities and providers can be different depending on region, and may influence the ability to translate study results to other healthcare environments. On the other hand, the highly flexible and pragmatic design of integrative care in this trial may actually enhance its generalizability. This 'real-world' approach has been identified as a design option for phase III trials of complex interventions, which may have some advantage over a tightly standardized care plan. Of note, a deliberate description of elements and characteristics of the intervention are important for drawing conclusions about the intervention, and its application to a variety of clinical settings[43]. It is important to acknowledge that the results of this trial will provide information on the efficacy of this specific model of integration only. While it does not represent the effectiveness of all integrative models in aggregate, this study does provide an important foundation for the development and implementation of future integrative models for back pain care.

From a delivery perspective, the financial feasibility of this or similar integrative care models must be considered alongside cost-effectiveness of patient outcomes. The breadth and scope of disciplines, weekly care team meetings, and facilitation by case managers resulted in a resource-intensive intervention. If clinical outcomes are positive, future investigation should explore which are the most essential elements of the pathway outlined in this study. Lessons from this study suggest established integrative teams, whose members can provide multiple types of therapy, access common patient information via electronic record systems, and anticipate responders to types of care, would likely contribute to financially viable models.

## Conclusion

This clinical care pathway was a useful tool for the consistent application of evidence-based healthcare. Exploring these models could be essential to improving the management of chronic low back pain, especially in the context of multidisciplinary or integrative care settings.

## Competing interests

The authors declare that they have no competing interests.

## Authors' contributions

All authors have contributed significantly to the design of the care pathway used in this clinical trial. GB is the principal investigator of the HRSA grant award. GB and MM are co-lead investigators for project implementation. MM, KW, and CL prepared the first draft of the manuscript and MM organized revisions. KW was a member of the integrative care team. CL was a case manager and meeting facilitator. All authors read and approved the final manuscript.

## Pre-publication history

The pre-publication history for this paper can be accessed here:

http://www.biomedcentral.com/1472-6963/10/298/prepub
